# Use of antidepressants and risks of restless legs syndrome in patients with irritable bowel syndrome: A population-based cohort study

**DOI:** 10.1371/journal.pone.0220641

**Published:** 2019-08-01

**Authors:** Yung-Chu Hsu, Hsin-Yi Yang, Wan-Ting Huang, Solomon Chih-Cheng Chen, Herng-Sheng Lee

**Affiliations:** 1 Division of Neurology, Department of Internal medicine, Ditmanson Medical Foundation Chia-Yi Christian Hospital, Chia-Yi City, Taiwan; 2 Clinical Medicine Research Center, Ditmanson Medical Foundation Chia-Yi Christian Hospital, Chia-Yi City, Taiwan; 3 Department of Pediatrics, School of Medicine, College of Medicine, Taipei Medical University, Taipei, Taiwan; 4 Department of Pediatrics, School of Medicine, College of Medicine, Kaohsiung Medical University, Kaohsiung, Taiwan; 5 Department of Pathology and Laboratory Medicine, Kaohsiung Veterans General Hospital, Kaohsiung, Taiwan; Fordham University, UNITED STATES

## Abstract

Previous research has suggested an association between antidepressants use and clinical restless legs syndrome (RLS) in patients, but there has never been a single study investigating the risk of RLS in irritable bowel syndrome (IBS) patients treated with antidepressants. Hence, we aimed to explore the association between IBS and RLS and to examine the risk of RLS in IBS patients treated with antidepressants. With the use of the National Health Insurance Research Database of Taiwan, 27,437 adults aged ≥ 20 years with newly diagnosed IBS (ICD-9-CM Code 564.1) and gender- and age-matched 54,874 controls without IBS were enrolled between 2000 and 2012. All patients were followed-up until RLS diagnosis, withdrawal from the National Health Insurance program, or end of 2013. We used the Cox proportional hazards model to calculate the hazard ratios (HRs) and 95% confidence intervals (CIs) of RLS. RLS was more prevalent in IBS patients than in the non-IBS group (7.57 versus 3.36 per 10,000 person-years), with an increased risk of RLS (adjusted HR [aHR], 1.91; 95% CI, 1.52–2.40). Multivariate Cox proportional hazards analysis identified older age (age, 51–65 years; aHR, 1.67; 95% CI, 1.09–2.56; and age > 65; aHR, 1.59; 95% CI, 1.02–2.48), hypothyroidism (aHR, 4.24; 95% CI, 1.92–9.37), CAD (aHR, 1.70; 95% CI, 1.17–2.48), and depression (aHR, 3.15; 95% CI, 2.14–4.64) as independent RLS risk factors in IBS patients. In addition, the male SSRIs users were associated with significantly higher risk of RLS (aHR, 3.05 95% CI, 1.34–6.92). Our study showed that the IBS group has higher risk of RLS. Moreover, SSRIs use may increase the risk of RLS in male IBS patients.

## Introduction

Irritable bowel syndrome (IBS) is a chronic, relapsing gastrointestinal disorder characterized by recurrent abdominal discomfort or pain, bloating, abnormal stool form and frequency, straining at defecation, and urgency [1]. The global pooled prevalence of IBS has been estimated to be ranging from 9%–23%, with a female predominance [2]. The pathophysiology of IBS is poorly understood and multiple factors are involved in the pathogenesis of IBS, including genetic susceptibility, visceral hyperalgesia, altered gastrointestinal motility, and neurological and psychological factors [3, 4]. Moreover, changes in the small intestinal bacterial overgrowth (SIBO)-related inflammatory mediator impaired communication between enteric and central nervous system are reported as the pathogenetic mechanisms [5]. A couple of studies have suggested that sleep disorders, particularly restless legs syndrome (RLS), are possible comorbidities in IBS patients [6, 7]. According to the previous studies, nearly 25%–30% of IBS patients experience RLS [6–8].

RLS is a common sensorimotor disorder characterized by intense restlessness and unpleasant creeping sensations deep inside the lower legs [9] and it is associated with SIBO [10]. The pathophysiology of RLS and IBS are yet to be fully elucidated. Inflammatory, immune and autonomic functions, and psychosocial status are the common causative factors of RLS and IBS. The brain–gut axis is an intricate bidirectional system which regulates the interactions between the brain and the gastrointestinal tract and may be the common basis of these diseases [11].

Antidepressants, such as tricyclic antidepressants (TCAs) and selective serotonin reuptake inhibitors (SSRIs), are effective in managing IBS symptoms [12]. These antidepressants may reduce IBS symptoms through different mechanisms. For instance, these antidepressants may change the patient’s perceptions of pain by modulating the visceral afferents centrally, treating the comorbid psychological symptoms, and altering the intestinal motility [13]. A meta-analysis indicated that TCAs and SSRIs improve IBS symptoms [14, 15]. However, these medications are associated with an increased incidence of RLS [16], most probably caused by enhanced serotonin and norepinephrine functions and inhibited dopaminergic activity.

At present, there is no large-scale and population-based study to evaluate the increased risk of RLS in IBS patients. Moreover, there has never been a single study investigating the risk of RLS in IBS patients treated with antidepressants. Herein, this study aimed to explore the association between IBS and RLS and to examine the risk of RLS in IBS patients treated with antidepressants by using the National Health Insurance (NIH) Research Database (NHIRD) in Taiwan.

## Patients and methods

### Data sources

This retrospective cohort study was conducted by using data from NHIRD in Taiwan. The NHI program in Taiwan was instituted in 1996 and is a single-payer universal insurance plan. Until 1998, nearly 99% of the 23 million citizens in Taiwan were enrolled. The NHIRD included the demographic data of enrollees, service records and expenditure claims from outpatient, inpatient and ambulatory care, and data associated with contracted pharmacies for reimbursement purposes. International Classification of Disease, 9th Revision, Clinical Modification (ICD-9-CM) was used to define the disease in this database. In the present study, data were obtained from the Longitudinal Health Insurance Database (LHID) 2005, a subset of the NHIRD. The LHID 2005 consists of all the original medical claims for 1,000,000 enrollees’ historical ambulatory data and inpatient care data under the Taiwan NHI program from 1997 to 2013, and the database was created and publicly released to researchers. No statistically significant differences exist in age, sex, or health care costs between the LHID 2005 sample group and all enrollees according to a National Health Research Institutes report [17].

This study was reviewed and approved by the Institutional Review Board of the Ditmanson Medical Foundation Chia-Yi Christian Hospital, Taiwan (CYCH-IRB No. 106023). All patient data from NHIRD were anonymized; therefore, the board did not require informed consent from the patients for this study.

### Study population

By using the data extracted from the LHID 2005, we conducted a retrospective cohort study of patients aged ≥20 years who were newly diagnosed with IBS (ICD-9-CM: 564.1) between 2000 and 2012, and were included in the IBS group [18, 19]. The index date was defined as the date of the first recorded diagnosis of IBS. In addition, each enrolled patient was required to have made at least three outpatient visits and only those patients whose diagnoses were not altered within 3 months of the index date were recruited [20]. To ensure the patients were newly diagnosed with IBS, we excluded patients who were diagnosed with IBS between 1997 and 1999. Subjects without IBS were randomly selected from the LHID 2005 and included in the non-IBS group. The subjects in the non-IBS group were frequency-matched with the IBS cohort at an approximately 2:1 ratio for age, sex, and index year of diagnosing IBS.

### Antidepressant assessment

Anatomical Therapeutic Chemical classification system-based pharmacological coding system was used in this study. Antidepressants were identified as N06A. In the present study, antidepressants were classified as TCAs (e.g., citalopram, escitalopram, fluoxetine, fluvoxamine, paroxetine, and sertraline), SSRIs (e.g., citalopram, escitalopram, fluoxetine, fluvoxamine, paroxetine, and sertraline) and other antidepressants (e.g., duloxetine, milnacipran, venlafaxine mirtazapine, and moclobemide). Information on the antidepressant exposure of IBS patients was confirmed with the prescription claims in the NHIRD. Each patient’s antidepressant exposure was determined using the cumulative dose of antidepressants, which is quantified by a defined daily dose as defined by World Health Organization. A defined daily dose value of less than 28 was considered nonuse.

### Outcome

The study primary outcome was the occurrence of RLS (ICD-9-CM: 333.90 and 333.99) during the follow-up period. The secondary outcomes were to investigate the risk of RLS in IBS patients treated with antidepressants. In addition, gender-based subgroup differences in treatment with antidepressants were examined. RLS diagnosis complied with the recommendations of the International RLS Study Group. All participants were observed until they were diagnosed with RLS or death, withdrawal from the NHI system, or December 31, 2013.

### Baseline characteristics and comorbidities

The general characteristics of the individuals were comprised of age, gender, insurable salary (in New Taiwan Dollars [NT$]; <19,100, 19,100–41,999, and ≥42,000), and urbanization level of residence (levels 1–4). The comorbidities consisted of common medical diseases and disorders that may possibly affect IBS or RLS [21, 22]. The covariates of comorbidities selected in this study, including hypertension (ICD-9-CM: 401–405), dyslipidemia (ICD-9-CM: 272), stroke (ICD-9-CM: 430–438), hyperthyroidism (ICD-9-CM: 242), hypothyroidism (ICD-9-CM: 244), chronic kidney disease (ICD-9-CM: 580–587), coronary artery disease (CAD; ICD-9-CM: 410–414), diabetes mellitus (ICD-9-CM: 250) and depression (ICD-9-CM: 296.2–296.3, 300.4, and 311), were defined as the diseases diagnosed before the index date.

### Statistical methods

All statistical analyses were performed using SPSS for Windows version 21.0 (IBM Corp., Armonk, NY, USA). Statistical graphs were plotted with R version 3.5.1, with the KMsurv, survfit, and survival packages. A two-tailed *p*-value of < 0.05 was considered statistically significant. Basic information such as age was expressed as mean ± standard deviation, whereas sex, baseline comorbidity, and income level were presented as number and percentage. Continuous variables were compared using t‐test and categorical variables using chi‐squared test or Fisher’s exact test, as appropriate. The incidence rate was calculated as the number of first diagnoses of RLS per 10,000 person-years. Hazard ratios (HRs) and 95% confidence intervals (CIs) for developing RLS were calculated using univariate and multivariate Cox proportional hazards models. Multivariable Cox proportional hazards models were used to analyze the association between antidepressant use and risk of RLS, adjusting for age, gender, and medical comorbidities. The cumulative risks of RLS for the IBS and non-IBS groups were measured using the Kaplan–Meier analysis method. Comparison of the two survival curves was performed using the log-rank test. Subgroup analyses by gender were used to find any potential differences in the response to antidepressants.

## Results

### Subject characteristics

This study involved a total of 27,437 IBS patients and 54,874 controls (Table 1). The mean ages of the IBS patients and controls were both 51.89 ± 16.56 years old. Males represented 49.71% and females 50.29% of the entire study population. The IBS group had higher prevalence of listed comorbidities and higher income level than the non-IBS group (*p* < 0.05).

**Table 1:** Baseline demographic factors and comorbidity of the study participants.

	IBS Group	Non-IBS Group	*p*-value
	N = 27,437	N = 54,874	
Age	51.89 ± 16.56	51.89 ± 16.56	0.948
≤50	13,156 (47.95)	26,361 (48.04)	
51–65	6,921 (25.23)	13,786 (25.12)	
>65	7,360 (26.83)	14,727 (26.84)	
Gender			1.000
Female	13,797 (50.29)	27,594 (50.29)	
Male	13,640 (49.71)	27,280 (49.71)	
Comorbidity			
Hypertension	8,544 (31.14)	13,720 (25.00)	<0.001
Dyslipidemia	5,846 (21.31)	7,724 (14.08)	<0.001
Stroke	2,629 (9.58)	4,175 (7.61)	<0.001
Hyperthyroidism	745 (2.72)	830 (1.51)	<0.001
Hypothyroidism	271 (0.99)	302 (0.55)	<0.001
CKD	1,771 (6.45)	2,627 (4.79)	<0.001
CAD	4,827 (17.59)	6,101 (11.12)	<0.001
DM	4,029 (14.68)	6,370 (11.61)	<0.001
Depression	1,946 (7.09)	1,453 (2.65)	<0.001
Income level			<0.001
Low	13,731 (50.05)	28,627 (52.17)	
Intermediate	11,206 (40.84)	21,414 (39.02)	
High	2,500 (9.11)	4,833 (8.81)	
Medications			<0.001
Nonuse	25,218 (91.91)	50253 (91.58)	
SSRIs	637 (2.32)	577 (1.05)	
TCAs	950 (3.46)	3134 (5.71)	
Other antidepressants	345 (2.26)	387 (0.71)	
≥2 antidepressants	287 (1.05)	523 (0.95)	

Data are presented as mean ± SD or number (percentage, %); CKD = chronic kidney disease; CAD = coronary artery disease; DM = diabetes mellitus.

### Incidence rate of RLS in the IBS and non-IBS groups

**Fig 1:**
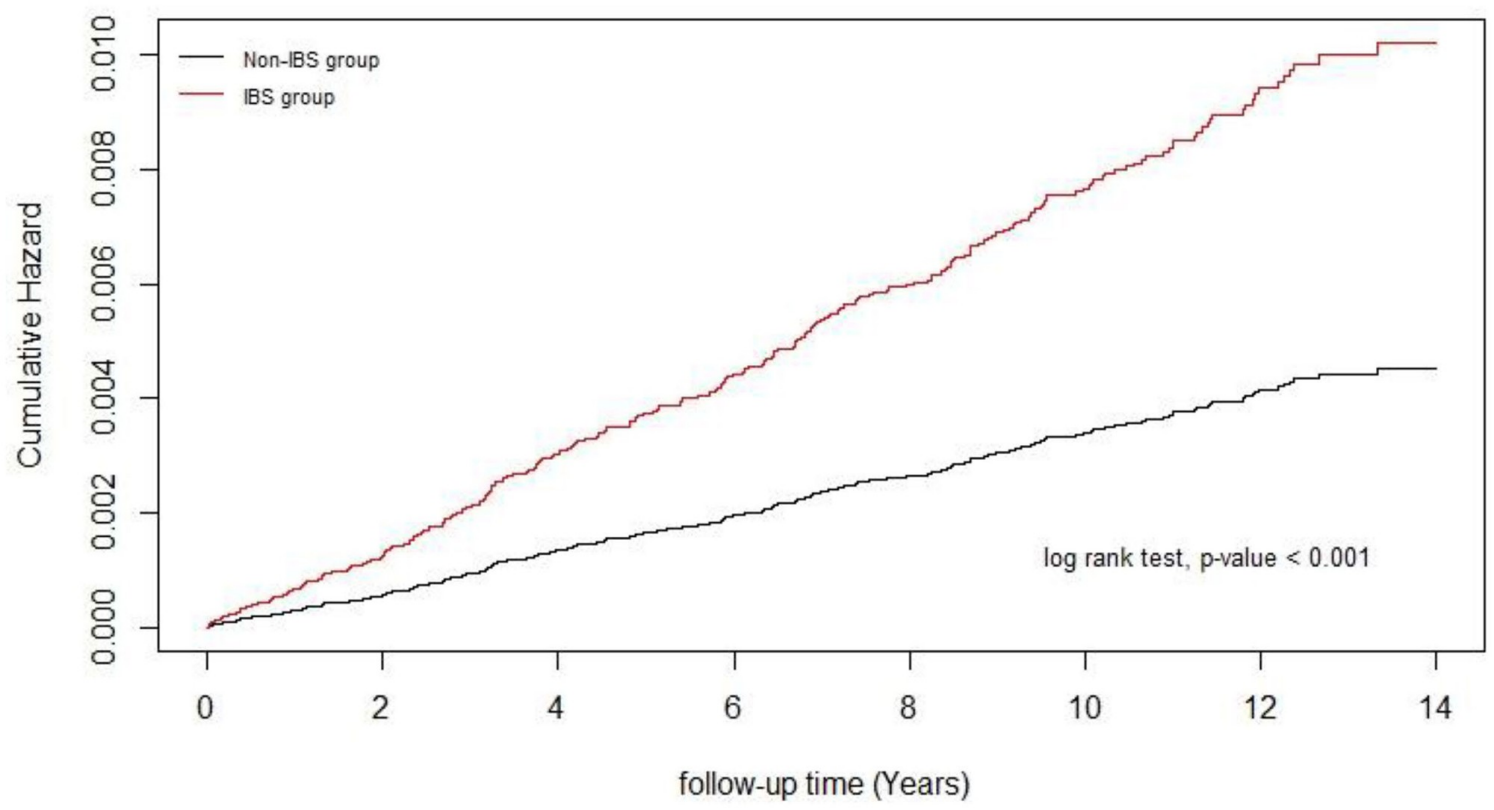
Cumulative incidence of RLS in the IBS and non-IBS groups, compared with a log-rank test. A statistically significant difference exists between the IBS and non-IBS groups (*p*<0.001).

The results of the log-rank test and cumulative incidence curve of RLS, as shown in Fig 1, revealed that the incidence of RLS in the IBS group was significantly higher than in the non-IBS group (log-rank test, *p* < 0.001). After adjustment for age, gender, comorbidity, and income level, the IBS group exhibited a 1.91-fold (95% CI, 1.52–2.39) higher risk of developing RLS than the non-IBS group. A multivariate Cox proportional hazards analysis identified that patients aged > 65 years (adjusted HR [aHR], 1.59; 95% CI, 1.17–2.16), hypothyroidism (aHR, 2.51; 95% CI, 1.16–5.46), CAD (aHR, 1.43; 95% CI, 1.06–1.92), and depression (aHR, 3.73; 95% CI, 2.74–5.10) were associated with significantly higher risk of RLS compared with patients without these conditions (Table 2).

**Table 2:** Cox model measured HRs and 95% CIs of the RLS associated with IBS and covariates.

Variables		Event	Person-years	IR	HR (95% CI)	
					Crude	Adjusted^&^
IBS	- No	147	437,265.74	3.36	1.00	1.00
	- Yes	165	218,078.92	7.57	2.27 (1.81–2.83)***	1.91 (1.52–2.39)***
Age	- ≤50	103	318,564.79	3.23	1.00	1.00
	- 51–65	79	160,610.09	4.92	1.52 (1.14–2.04)**	1.30 (0.95–1.78)
	- >65	130	176,169.77	7.38	2.28 (1.76–2.96)***	1.59 (1.17–2.16)**
Gender	- Female	168	331,286.18	5.07	1.00	1.00
	- Male	144	324,058.47	4.44	0.88 (0.70–1.10)	0.94 (0.75–1.19)
Comorbidity						
Hypertension	- No	179	490,169.09	3.65	1.00	1.00
	- Yes	133	165,175.56	8.05	2.21 (1.77–2.77)***	1.28 (0.96–1.70)
Dyslipidemia	- No	237	565,859.12	4.19	1.00	1.00
	- Yes	75	89,485.54	8.38	2.03 (1.56–2.63)***	1.21 (0.90–1.62)
Stroke	- No	266	606,819.84	4.38	1.00	1.00
	- Yes	46	48,524.82	9.48	2.17 (1.59–2.97)***	1.09 (0.77–1.54)
Hyperthyroidism	- No	303	645,028.65	4.70	1.00	1.00
	- Yes	9	10,316.00	8.72	1.87 (0.96–3.63)	1.28 (0.65–2.54)
Hypothyroidism	- No	305	651,740.50	4.68	1.00	1.00
	- Yes	7	3,604.16	19.42	4.19 (1.98–8.87)***	2.51 (1.16–5.46)*
CKD	- No	294	624,952.36	4.70	1.00	1.00
	- Yes	18	30,392.29	5.92	1.27 (0.79–2.04)	0.72 (0.44–1.18)
CAD	- No	230	576,203.88	3.99	1.00	1.00
	- Yes	82	79,140.78	10.36	2.61 (2.03–3.36)***	1.43 (1.06–1.92)*
DM	- No	253	581,536.82	4.35	1.00	1.00
	- Yes	59	73,807.84	7.99	1.85 (1.39–2.45)***	1.07 (0.78–1.47)
Depression	- No	260	632,280.42	4.11	1.00	1.00
	- Yes	52	23,064.24	22.55	5.53 (4.11–7.46)***	3.73 (2.74–5.10)***
Income level	- Low	169	330,222.55	5.12	1.00	1.00
	- Intermediate	126	266,816.21	4.72	0.92 (0.73–1.16)	0.87 (0.69–1.10)
	- High	17	58,305.90	2.92	0.57 (0.35–0.94)*	0.61 (0.37–1.01)

IR = incidence rate per 10,000 person-years; CI = confidence interval; HR = hazard ratio.

***p < 0.001;

** *p* < 0.01;

*p < 0.05.

^&^Adjusted for age, gender, hypertension, dyslipidemia, stroke, hyperthyroidism, hypothyroidism, CKD, CAD, DM, depression, and income level.

### The risk factors of RLS in the IBS group

Multivariate Cox proportional hazards analysis identified older age (age, 51–65 years; aHR, 1.67; 95% CI, 1.09–2.56; and age > 65; aHR, 1.59; 95% CI, 1.02–2.48), hypothyroidism (aHR, 4.24; 95% CI, 1.92–9.37), CAD (aHR, 1.70; 95% CI, 1.17–2.48), and depression (aHR, 3.15; 95% CI, 2.14–4.64) as independent RLS risk factors in IBS patients. In addition, after controlling the confounding factors, SSRIs and TCAs use were associated with significantly increased risk of RLS (aHR, 2.49; 95% CI, 1.21–5.12; and aHR, 1.52; 95% CI, 1.03–2.24, respectively; Table 3).

**Table 3:** The cox proportion hazard regression model of RLS in IBS patients.

	Crude HR (95% CI)	*p*-value	Adjusted HR (95% CI)^&^	*p*-value
Age				
≤50	1.00		1.00	
51–65	1.98 (1.33–2.94)	0.001	1.67 (1.09–2.56)	0.019
>65	2.47 (1.71–3.57)	<0.001	1.59 (1.02–2.48)	0.039
Gender				
Female	1.00		1.00	
Male	0.87 (0.64–1.19)	0.384	0.97 (0.71–1.32)	0.832
Comorbidity				
Hypertension	2.16 (1.59–2.93)	<0.001	1.26 (0.86–1.85)	0.234
Dyslipidemia	1.67 (1.18–2.37)	0.004	1.06 (0.73–1.56)	0.750
Stroke	2.31 (1.55–3.43)	<0.001	1.31 (0.84–2.02)	0.230
Hyperthyroidism	1.32 (0.54–3.23)	0.536	0.93 (0.37–2.34)	0.875
Hypothyroidism	5.49 (2.57–11.70)	<0.001	4.24 (1.92–9.37)	<0.001
CKD	1.16 (0.63–2.14)	0.636	0.76 (0.40–1.41)	0.381
CAD	2.67 (1.93–3.68)	<0.001	1.70 (1.17–2.48)	0.006
DM	1.50 (1.02–2.22)	0.041	0.90 (0.59–1.38)	0.642
Depression	3.95 (2.71–5.76)	<0.001	3.15 (2.14–4.64)	<0.001
Income level				
Low	1.00		1.00	
Intermediate	0.87 (0.63–1.20)	0.392	0.83 (0.60–1.14)	0.314
High	0.55 (0.28–1.09)	0.087	0.57 (0.28–1.16)	0.166
Medications				
Non use	1.00		1.00	
SSRIs	2.59 (1.26–5.32)	0.010	2.49 (1.21–5.12)	0.013
TCAs	1.73 (1.18–2.55)	0.006	1.52 (1.03–2.24)	0.036
Other antidepressants	2.17 (0.88–5.33)	0.091	1.90 (0.77–4.67)	0.164
≥2 antidepressants	3.59 (2.24–5.77)	<0.001	3.30 (2.05–5.32)	<0.001

^&^Adjusted for age, gender, hypertension, dyslipidemia, stroke, hyperthyroidism, hypothyroidism, CKD, CAD, DM, depression, and income level.

### Stratification by gender group for the risk of RLS in terms of antidepressant users with IBS

Table 4 shows the gender stratification analysis of the risk of RLS in terms of IBS patients with antidepressant use. For SSRIs male users, the aHRs was 3.05, (95% CI, 1.34–6.92) as compared with non-SSRIs male users. We demonstrated that male IBS patients with or without SSRI use had a higher risk of RLS than males in the non-IBS group (*p* < 0.001, S1 Table). In the male group, the other antidepressants users were associated with significantly higher risk of RLS (aHR, 3.59; 95% CI, 1.37–9.40). No significant difference in the risk of RLS was found between the antidepressant and non-antidepressant female users.

**Table 4:** Gender-stratified multiple Cox proportional hazards regression analysis of the RLS risk in IBS patients with antidepressant use.

	Male		Female	
	^&^Adjusted HR (95% CI)	*p*-value	^&^Adjusted HR (95% CI)	*p*-value
Drug				
Nonuse	1.00		1.00	
SSRIs	3.05 (1.34–6.92)	0.008	1.31 (0.47–3.62)	0.609
TCAs	1.89 (0.81–4.44)	0.141	0.84 (0.34–2.09)	0.706
Other antidepressants	3.59 (1.37–9.40)	0.009	1.82 (0.56–5.94)	0.318
≥2 antidepressants	0.99 (0.13–7.32)	0.992	1.02 (0.25–4.23)	0.918

^&^Adjusted for age, hypertension, dyslipidemia, stroke, hyperthyroidism, hypothyroidism, CKD, CAD, DM, depression, and income level.

## Discussion

This nationwide population-based cohort study indicated IBS is a potential risk factor for developing RLS, even after adjusting for age, gender, income, urbanization and comorbidities. Additionally, we found that SSRIs use was associated with an increased risk of RLS in male IBS patients. Our results are consistent with previous studies showing an association between antidepressant use and significantly higher risk of RLS in IBS patients [6–8, 23]. However, these studies are small-scale, cross-sectional, or case-control studies. The design of this study may provide a more objective evaluation on the association between IBS and RLS risk. The present study utilizes a large population-based dataset and nation-based investigation to minimize any possible surveillance bias, hence making our research more generalizable to the general population. Moreover, this study considered possible confounding factors of RLS.

In the current study, consistent with previous observations [21], the possible comorbidities of RLS, such as hypothyroidism, CAD, and depression were more prevalent in the IBS group than in the comparison group. To date, the association between thyroid disorders and RLS is not known. Yet, our findings show a higher risk of hypothyroidism in RLS patients. The occurrence of RLS might be caused by an imbalance between the dopaminergic agonist system and thyroid hormones [21, 24]. Moreover, we found a higher risk of RLS with CAD in this research. The possible explanation of higher RLS risk may be due to the decrease of cardiovascular baroreflex gain and greater peripheral vascular resistance inspected in CAD patients. A study reported that RLS can directly contribute to changes in the cardiovascular system autonomic control, regardless of differences in the sleep quality [25]. The association between RLS and depression is well known and the findings of the current study are consistent with previous observations [26]. Epidemiological studies reported that most individuals suffering from RLS above middle age. Our findings are also consistent with epidemiological studies stating that the prevalence of RLS increases with age [27, 28]. The findings of our study are in agreement with the earlier documentation of these conditions as potential risk factors for RLS.

The exact pathophysiologic mechanisms linking IBS and RLS are unknown. Their etiologies are most likely multifactorial, involving both biological and psychosocial factors. There are several possible explanations for the association between IBS and RLS risk. Previous studies have identified SIBO and several sensory disorders, including RLS, as possible contributing factors of IBS [29]. A study showed that RLS patients have a higher SIBO rate (69%) than controls (28%) [10]. Another study showed that RLS patients are more likely to develop IBS than controls (28% versus 4%) [30]. Another previous study has also found that in patients with both SIBO and RLS, the RLS symptoms improved after being treated for SIBO [29]. An additional double-blind, placebo-controlled study also reported that treatment of SIBO with antibiotic rifaximin significantly improved the RLS symptoms in patients with both conditions [31]. These studies reveal a causal link between SIBO and RLS. In addition, IBS is associated with systemic inflammation, immune alterations, and SIBO. Immune dysfunction might play a more general role in RLS [30]. As further evidence, the neutrophil counts and neutrophil-to-lymphocyte ratio are significantly higher in RLS patients [32] and elevated blood level of C-reactive protein is associated with an increased RLS severity [33].

In addition, genetic predisposition increases the risk of SIBO in RLS patients [34]. The *PTPRD*, *BTBD9*, *MAP2K5*, *MEIS1*, *SKOR1*, and *TOX3* genes are suggested to influence neurodevelopmental processes, iron metabolism [35], and dopamine systems [36, 37]. This may explain some of the iron or dopamine changes in relation to RLS. Iron is required for proper dopamine signaling; evidence suggests that disrupted brain iron trafficking leads to disturbances in the striatal dopamine neurotransmission [35]. SIBO inflammation leads to increased hepcidin and central nervous system iron deficiency, which, in turn, leads to RLS. Generally, studies suggest that RLS may be related to abnormalities in the central dopamine pathways [38, 39].

Another possible pathogenetic mechanism is vitamin D metabolism-related. Vitamin D may play a role in the pathophysiology of RLS by modulating the dopaminergic system. The available evidence suggests that low vitamin D status is common among the IBS population [40]. Therefore, it is possible that the increased risk of RLS is related to the gut irritation and inflammation caused by IBS, which thereby translate to poor absorption of nutrients [41, 42].

A few of population-based studies reported that European and North American populations show higher RLS prevalence than the Asian population [43–46]. In the present study, the annual incidence rate of RLS in non-IBS Taiwanese (3.36/10,000 person-years) is similar to that in non-IBS Chinese (3.42 and 3.76/10,000 person-years) [44, 45] but lower than that in non-IBS German (14–43/1,000 person-years) [46]. Numerous studies show that the prevalence of RLS is approximately twice as high in women than in men [43, 47]. However, RLS shows no clear sex-dependent predisposition in our study.

The current study evaluated that SSRIs use is associated with an increased risk of RLS in IBS patients. Our results are generally consistent with the findings of previous studies [48–51]. The association between the use of SSRIs and RLS can be explained by the roles of SSRIs in dopamine and serotonin signaling; they increase the serotonin levels and decrease dopamine production, which triggers the development of RLS [52, 53]. However, some studies concluded that there was no association between antidepressants and RLS. Brown et al. found no significant associations between RLS and the use of any specific class of antidepressant in patients with sleep disorders [49]. A case-control study reported that the use of SSRIs and TCAs was not an independent predictor of RLS [54]. Leutgeb et al. study indicated that antidepressants were not shown to be a major risk factor for RLS in patients with affective and anxiety disorders [48]. This inconsistency may be due to the heterogeneous study designs, sample selection criteria, or ethnic differences. Although, antidepressants have been shown to help relieve symptoms of IBS and are considered as a safe treatment for IBS [14]. The physicians should still aware of the possible negative effects of antidepressants.

Few researches have investigated the gender differences in the association between antidepressant use and RLS. Prior studies indicate that antidepressant use is more strongly associated with RLS in men than in women [55]. Another research showed that antidepressant-induced RLS has been most often observed in women and the elderly [49]. In the present study, we found that antidepressant male users have a higher risk of RLS, which is inconsistent with the results of previous studies. Although the grounds for the gender-specific association of antidepressant use and RLS is not clear. It may be related to differences in body weight, volume of plasma, gastric emptying and acid production, splanchnic blood flow, plasma protein levels, enzyme activity, as well as drug transport and clearance rate differences between sexes [56]. In addition, it might be related to the patient’s gender and type of antidepressant used. Previous study was found that fluoxetine was significantly associated with RLS in women than in men, citalopram, paroxetine, and amitriptyline are associated with RLS [55]. Although antidepressant medication has a controversial association to RLS with numerous reports that they aggravate RLS, no study to date has examined the risks of RLS in IBS patients treated with antidepressants. Our study pioneered the investigation and showed that antidepressant male users inclined to display higher risk of RLS. Future studies need to replicate and evaluate possible reasons for the observed gender differences.

### Strengths and limitations

The major strength of this study lies in its design, which allows a large sample size and sufficient statistical power to investigate the relationship between IBS and RLS and a variety of covariates. However, there are limitations in this study. First, it was difficult to assess the impact of diverse IBS subtypes and IBS severity on RLS in this study because the relevant clinical parameters of IBS were not included in the NHIRD dataset. Second, the NHIRD did not include any clinical information such as RLS severity, neuroimaging, or other laboratory results. Therefore, the precise diagnosis of idiopathic or secondary RLS was not available in this database. Finally, important information on psychological status, sleep quality, and individual behavior were not recorded in the NHIRD. These factors may influence the RLS risk.

## Conclusions

In conclusion, this nationwide population-based study shows that the IBS group has increased risk of RLS. Moreover, SSRI use may increase the risk of RLS in male IBS patients. Further investigation is necessary to examine both the mechanism and causality of this relationship.

## Supporting information

S1 Table Adjusted HRs measured using multiple Cox proportional model for the male RLS patients associated with IBS and SSRI supplementation. (DOCX)
