# Supplementary material for: Use of antidepressants and risks of restless legs syndrome in patients with irritable bowel syndrome: A population-based cohort study
Source: PLoS One. 2019 Aug 1;14(8):e0220641. doi: 10.1371/journal.pone.0220641 (PMC6675099; doi:10.1371/journal.pone.0220641)
Supplement: S1 Table — (DOCX) [file pone.0220641.s001.docx]

Table S1. Adjusted HRs measured using multiple Cox proportional model for the male RLS patients associated with IBS and SSRI supplementation.

| IBS | SSRIs | Crude HR  (95% CI) | *p-*value | Adjusted HR  (95% CI)^&^ | *p-*value | *p* for trend |
| --- | --- | --- | --- | --- | --- | --- |
| - | - | 1.00 |  | 1.00 |  | <0.001 |
| - | + | 2.12 (1.50–3.00) | <0.001 | 1.82 (1.28–2.58) | 0.001 |  |
| + | - | 3.86 (1.41–10.60) | 0.009 | 2.53 (0.91–7.03) | 0.075 |  |
| + | + | 7.19 (3.79–13.63) | <0.001 | 4.10 (2.08–8.06) | 0.004 |  |

^&^Adjusted for age, gender hypertension, dyslipidemia, stroke, hyperthyroidism, hypothyroidism, CKD, CAD, DM, depression, and income level.
